# Notes and News

**Published:** 1888-03-24

**Authors:** 


### NOTES AND NEWS.

Collected and Communicated.

Dr. David Shirres has resigned his post as senior resident medical officer to the Aberdeen Infirmary.

At the festival dinner of the Jewish Hospital and Orphan Asylum subscriptions to the amount of nearly £5,000 were announced.

We are compelled to hold over our account of the interesting paper on Lying-In Hospitals, by Mr. Ryan, the Secretary of St. Mary's Hospital, and the discussion which followed it, until next week.

Mr. R. J. Shepard, who has been for many years treasurer of the Royal Surrey County Hospital, has resigned his post, which has been accepted by Mr. Budgett. At the Annual Court of Governors of the Hospital the Rev. Dr. Robson moved that the hearty thanks of the Governors should be given to Mr. Shepard for his unwearied devotion to the hospital during the time he has been its treasurer.

The finances of the Gordon Hospital for Fistula, &c., Vauxhall Road, are in a somewhat unfortunate condition, the committee having been compelled to borrow money for pressing liabilities. The debt of the hospital in this, the fourth year of its existence, stands at £700. The receipts for the year amounted to £867 8*s.* 2*d.*, and the expenditure to £9,358 11*s.* 7*d.*, leaving a large deficit, although most of the in-patients paid a small sum—from 5*s.* to £3 3*s.* a-week—for their treatment. On the other hand, the hospital can show a good record of work done, and when its usefulness is better known to the public it will doubtless receive a larger measure of support.

The report of the Delancey Fever Hospital, Cheltenham, is an eminently satisfactory document. It records that the town has been comparatively free from the infectious diseases which have been prevalent in many parts of the kingdom during 1887. Two distinct importations of small-pox—one from Bristol and the other from Dublin—occurred, but, thanks to the precautions taken, and to the isolation afforded, there was no further spread of infection. "Thus for another year," says the Report, "the town has been saved from the risks of a small-pox epidemic, and for fourteen years—during which there have been twenty-six distinct importations, forty-one cases, and five deaths—there has in no single instance been a spread of the disease, and Cheltenham has enjoyed an immunity such as can be claimed for no other town of its size in the kingdom." An equally satisfactory record is given of the other less infectious diseases with which the Delancey Hospital deals.

Hospitals receive money from strange sources. The secretary of the Bristol Royal Infirmary acknowledges the receipt of £3 3*s.*, the proceeds of a baby-show recently held in the town.

The cost of the in-patient department at the Manchester Hospital for Consumption has during the last year averaged 19*s.* 6*d.* per head weekly, as against 22*s.* at Ventnor, and from 30*s.* to 35*s.* at Brompton.

Mrs. Rusher has presented property to the value of £3,000 to the fund for establishing a convalescent home for the Metropolitan and City Police. This property consists of two houses at Dover, inter-communicating with each other, and provided with beds, bedding, and furnishings of every kind. The fund now amounts to £9,000, exclusive of Mrs. Rusher's munificent gift.

At a conference of delegates of the Ancient Order of Foresters, held at the Market Hotel, Birmingham, it was stated that the report of the Foresters' Convalescent Home at Clent for the first year of its existence showed a balance in hand of £115 3*s.* 9*d.* after all expenses were paid. The number of members who have subscribed to the home is 16,686. This is a friendly society that proves itself worthy of the name.

Miss S. Barchard writes to the *Yorkshire Post*, asking the readers of that newspaper to send her old books for the purpose of forming a library at a workhouse hospital where there are 800 patients. It might with advantage be remembered oftener than it is that it would be a great boon to the sick poor in workhouses to share in some of the little comforts which brighten the long hours to the patients in other hospitals.

In one hospital there have been treated in the course of a year nearly 3,000 children who were suffering from the effects of insufficient food and exposure to cold. It is to help these that the Poor Children's Aid Society exists. A large proportion of the children of the poor go to school without having tasted food, a still larger number are insufficiently clothed; this society strives to help both of these classes. It gives them food and lends them clothes. This lending is pretty much a formal business; but while there are parents who regard all things as good to take to the pawnshop, it is necessary for the Society to retain some claim upon their gifts.

The Governors of the Bridport Dispensary are trying to reduce the number of patients profiting by it, as the state of its finances demands the exercise of the strictest economy. But it appears that the rules of the dispensary have been either evaded or forgotten. Two of the classes for whom it is *not* meant are "members of a sick club" and "domestic servants in place," and both of these have taken advantage of it; aided, of course, by subscribers, who give away three times as many letters of recommendation as their subscription entitles them to. If these abuses were rectified, it is probable that the funds of the dispensary would be found quite sufficient for its expenses.

As a memorial of the Queen's Jubilee, the Committee of the Suffolk General Hospital appealed to the public for funds, to be expended in "facing the walls of the two lower wards with tiles, and forming a retiring pension fund for nurses." Two very good objects, which the public sympathised with to the extent of £487 2*s.* 6*d.* But which is the more important? Let each judge according to his lights. The lights possessed by the Committee of the Suffolk General Hospital led them to expend the money thus: Messrs. Simpson and Son, for their work in the wards, £451; postage, printing, advertising, etc., £31 0*s.* 5*d.*; leaving £5 2*s.* 1*d.* at Messrs. Gurney and Co.'s to the credit of the proposed nurses' pension fund. Alas, poor nurses!

THE following vacancies are announced this week, the amount of salary in each case being given after the name of the institution :—

**Resident Medical Officers.**

Borough Asylum, Birmingham. Clinical Assistant.—Board and residence.
British Seaman's Hospital, Cronstadt, St. Petersburg. Resident Medical Officer.—£180 per annum, with furnished apartments.
East London Hospital for Children, Shadwell, E. Resident Clinical Assistant.—Board.
General Infirmary, Northampton. House Surgeon.—£125 per annum, with board.
Hospital for Consumption and Diseases of the Chest. Resident Clinical Assistants.
Liverpool Dispensaries. Two Assistant Surgeons.—£80 per annum, with board, etc.
Ross-shire, Parish of Resolis and District. Resident Medical Officer.—£62 per annum.
Royal London Ophthalmic Hospital, Moorfields, E.C. Junior House Surgeon.—£50 per annum.
St. Helen's Friendly Societies' Medical Aid Association. Resident Medical Officer.
West Derby Union Workhouse, Walton on-Hill. Resident Assistant Medical Officer.—£100 per annum, with board.

**Non-resident Medical Officers.**

Charing Cross Hospital. Assistant Surgeon.
Charing Cross Hospital. Surgical Registrar.
Edenderry Union. Medical Officer, Carberry Dispensary.—£135 per annum, and fees.
St. Peter's Hospital for Stone, etc., Henrietta Street, W.C. Anæsthetist.—£50 per annum.
West London Hospital, Hammersmith Road. Clinical Assistants.
Westminster Hospital. Medical Registrar.—£40 per annum.

**Nurses.**

Monkwearmouth Dispensary, Sunderland. One, Trained Surgical.—£16.
Kingston Nurses' Home, Baker Street, Hull. Several, Monthly, Surgical, and Massage.
Nursing Institution, Burton-on-Trent. One, Medical and Surgical.—£25.
Nottingham and Notts Nursing Institution. One, Medical, Surgical, and Monthly.
Workhouse Infirmary Association, 6, Adam Street, Adelphi. Several.
Victoria Hospital Convalescent Home, Margate. One, Trained.
Cancer Hospital (Free), Brompton, S.W. One, Thoroughly Trained.—£20.
Hospital for Consumption, Ventnor, I.W. One, Assistant Nurse. —£18.

**Probationer.**

The Cottage Hospital, Llandudno. One.

**Housekeeper.**

Bradford Infirmary, Yorkshire. One.—£50.

IT is so much a matter of course for hospitals and other charitable institutions to begin life under pecuniary difficulties that the *Beckenham Journal* thinks that the Cottage Hospital in that town may be congratulated on starting with a debt of only £400. We shall reserve our congratulations for a year, by which time we shall know whether this debt is to increase or diminish.

THERE seems to be some cause for anxiety in the Liverpool Workhouse. There have been complaints that the hospital is overcrowded, and it is also alleged that a letter addressed to an inmate was not delivered to him on its arrival. He left the workhouse without it, though he had been there when it came, and when he returned to make inquiries respecting it he could gain no information. As it contained money to take him back to his native country—Scotland—the negligence involved a decided hardship.

MR. LEVESON-GOWER said a very solemn thing at the annual meeting of the Royal Berks Hospital, and to give point to it, he stated that it was a quotation from a very great physician who practised in London many years ago. It is this piece of advice :—" Have nothing to do with specialists unless you are quite sure that you are suffering from the disease that he has made his study, for if you have not got that disease he will make you believe you have." Let us hope that neither Mr. Leveson-Gower nor the "great physician" spoke from experience, and also that neither of them is responsible for the grammatical construction of the sentence.

THE General Court of the Governors of Guy's Hospita has sanctioned the erection of a residential medical college upon a site immediately contiguous to the hospital.

THE finance report of the Swansea Eye Hospital shows that the receipts and expenditure are exactly the same—£270 17*s*.

A LEGACY of £1,000 has reverted to the Wolverhampton General Hospital by the death of Mrs. Edwards, widow of Dr. Edwards, of Tettenhall, and a bequest of the same amount has been left to the hospital by the late Mr. Crowther Smith.

THE hospital chapel at Ilford is being restored and enlarged, under the superintendence of Mr. J. M. Brooks, architect. This chapel was founded by Adeliza, Abbess of Barking, in the reign of Stephen. The chapel dates from the fifteenth century, and is a long, narrow structure, 100 feet in length by 20 feet in width.

A NEW hospital, of which Mr. Sydney Mitchell, of Edinburgh, is the architect, is to be added to the Royal Montrose Asylum (Sunnyside), the doctors and other officials of which are determined that it shall continue to deserve the name it has gained as one of the most perfect homes for the sick in mind to be found in the kingdom. The hospital, as seen in the plan, is a picturesque building, consisting of four pavilions, and will provide accommodation for fifty patients of each sex.

THE *Luton Times* complains that modern philanthropy has no notion of "doing good by stealth," while the idea of "blushing to find it fame" is the very last thing that would happen to its disciples, whom, therefore, it regards with severe scorn. True, O *Luton Times* ! but there is such a thing as emulation in noble deeds, which is more than mere vanity ; and to put the matter on the lowest ground, the sight of Mr. Brown's name on a subscription list is often a powerful inducement to Mr. Smith to add his own.

THE directors of the London Stereoscopic Company announce that on the closing, at the end of this month, of the third annual exhibition of photographs taken by amateurs, now being held at their gallery in Regent Street, the whole of the 4,000 photographs sent in for competition will, with the exception of the prize pictures, be distributed among various hospitals and charities. This affords hospital managers an opportunity, seldom granted, of decorating the bare walls of their wards.

A MEETING of workmen elected by their fellows at Elswick Works, to represent them as governors of the Royal Infirmary, was held in the Elswick Works Girls' School, Newcastle. The men connected with these works subscribed £756 for the medical charities of the city, of which £653 was allotted to the Infirmary. For every £10 subscribed to the Infirmary the men are entitled to elect a governor. The 66 men chosen for that office met, therefore, to learn from the Rev. W. Moore Ede the nature of their duties, which they purpose taking up with care and energy.

THE *Aberdeen Herald* bears strong testimony to the value of good nursing in a hospital. "The mass of people in Aberdeen," it says, "have hardly even yet begun to realise the inestimable value to the patients, as well as the vital consequence to the success of any hospital, of careful and skilled attention by trained nurses. Granted that, and mere direct medical superintendence practically sinks to a secondary place ; but without it the best medical superintendence possible will not suffice to secure either the comfort or the safety of the patient."

